# Eco-Friendly Polysaccharide-Based Synthesis of Nanostructured MgO: Application in the Removal of Cu^2+^ in Wastewater

**DOI:** 10.3390/ma16020693

**Published:** 2023-01-10

**Authors:** Nayara Balaba, Dienifer F. L. Horsth, Jamille de S. Correa, Julia de O. Primo, Silvia Jaerger, Helton J. Alves, Carla Bittencourt, Fauze J. Anaissi

**Affiliations:** 1 Chemistry Department, Universidade Estadual Do Centro-Oeste, Guarapuava 85040-080, Brazil; 2 Chimie des Interactions Plasma-Surface (ChIPS), Research Institute for Materials Science and Engineering, University of Mons, 7000 Mons, Belgium; 3 Laboratório de Materiais e Energias Renováveis, LABMATER/UFPR, Universidade Federal do Paraná—UFPR, Palotina 85950-000, Brazil

**Keywords:** adsorption, heavy metals, magnesium oxide, copper, drinking water

## Abstract

The present study described three synthesis routes using different natural polysaccharides as low-cost non-toxic fuels and complexing agents for obtaining MgO. Cassava starch, *Aloe vera* leaves (mainly acemannan) gel, and citric pectin powder were mixed with magnesium nitrate salt and calcined at 750 °C for 2 h. The samples were named according to the polysaccharide: cassava starch (MgO-St), citrus pectin (MgO-CP), and *Aloe vera* (MgO-Av). X-ray diffraction identified the formation of a monophasic periclase structure (FCC type) for the three samples. The N_2_ adsorption/desorption isotherms (B.E.T. method) showed an important difference in textural properties, with a higher pore volume (V_max_ = 89.76 cc/g) and higher surface area (SA = 43.93 m^2^/g) obtained for MgO-St, followed by MgO-CP (V_max_ = 11.01 cc/g; SA = 7.01 m^2^/g) and MgO-Av (V_max_ = 6.44 cc/g; SA = 6.63 m^2^/g). These data were consistent with the porous appearance observed in SEM images. Porous solids are interesting as adsorbents for removing metallic and molecular ions from wastewater. The removal of copper ions from water was evaluated, and the experimental data at equilibrium were adjusted according to the Freundlich, Langmuir, and Temkin isotherms. According to the Langmuir model, the maximum adsorption capacity (q_max_) was 6331.117, 5831.244, and 6726.623 mg·g^−1^ for the adsorbents MgO-St, MgO-Av, and MgO-CP, respectively. The results of the adsorption isotherms indicated that the synthesized magnesium oxides could be used to decrease the amount of Cu^2+^ ions in wastewater.

## 1. Introduction

The mining process presents many ores and reject concentrations, which contributes as a source of contamination to the environment through mine wastewater [1]. Rejects can be rich in toxic and bio-accumulative metallic elements, even far from the mining areas, as the contamination can be carried for miles [2]. In 2015, the Rio Doce estuary in Mariana (Minas Gerais, Brazil) was severely impacted by the breach of an iron mine tailing dam, causing major environmental damage. Although the tailing released from the breached dam was composed primarily of non-toxic minerals, the high Fe–oxy–hydroxide content may have promoted the chemical binding of metals and metalloids accumulated within the basin. As a result, after years, there is still evidence of trace metal contamination of As, Cd, Cr, Cu, Hg, Mn, Pb, Se, and Zn in the rivers and streams near the region. It is estimated that 663.2 km from the mining company, about 43 million m^3^ of iron mine tailing were released [3,4]. Among the heavy metals, copper is one of the most abundant pollutants in mining wastewater [5]. Besides mines, it can be released into the environment through agricultural processes, solid waste disposal, welding and electroplating processes, sewage treatment, plumbing, and electrical wiring materials [5]. According to WHO (1993) standards, the permissible limit of Cu^2+^ in drinking water is 1.3–2.0 mg·L^−1^ because, in high concentrations, it can cause serious health effects such as damaging the central nervous system, increasing blood pressure, Alzheimer’s disease, and Menkes syndrome [6].

Therefore, industrial wastewater must be treated to protect the environment before it is disposed of into water bodies. Contamination by water does not occur only through direct ingestion but by other means, such as consuming vegetables and fruits from areas irrigated with contaminated water, thus increasing health risks. Several methods for the decontamination of Cu ^2+^ ions from water have been reported. Among them are flotation [6], electrocoagulation [7], ion exchange [8], precipitation [9], and adsorption [10,11]. Adsorption is one of the most common techniques due to its simplicity, high efficiency, and low cost [5]. In this context, magnesium oxide (MgO) has attracted interest due to its low cost, environmental friendliness, and high capacity to adsorb metal ions [12], dyes [13,14], carbon dioxide [15], and phosphates [16]. However, commercial MgO powder, often obtained by the thermal decomposition of MgCO_3_ or Mg(OH)_2_, as a metal adsorber has drawbacks, such as a low specific surface area, irregular morphology, and grain size [17]. Considerable methodologies for the synthesis of MgO have been reported in the literature such as precipitation [13], the calcination process [17], and hydrothermal- and microwave-assisted methods [18]. In light of this, the search for natural additives for materials synthesis has recently gained importance due to their reported low environmental impact, higher chemical reactivity, high combustion power, reduced calcination temperature, and action as a complexing gelling agent [19]. Synthesis routes using natural additives are considered eco-friendly. For example, polysaccharides from plants can be used as fuel, thus replacing other types of synthetic reagents such as complexing agents (oxalic, tartaric, and acetic acid) and surfactants (including cetyltrimethylammonium bromide, ethanol, ethylene glycol, and glycerin) [20] that are widely used as modifying agents to obtain MgO particles. Polysaccharides act as low-cost, non-toxic alternative additives produced from natural plants, acting as complexing agents for the central metal ions and can tailor the growth of MgO crystals [21].

This study used three different polysaccharides in the MgO synthesis: starch, citrus pectin, and *Aloe vera*. Synthesis 1 used starch from cassava (*Manihot esculenta*), an abundant plant in Brazil. This biodegradable polysaccharide consists of amylose and amylopectin molecules composed of D-Glucose units [22]. In synthesis 2, citrus pectin was used, a polysaccharide from the peels of citrus fruits. This polysaccharide consists of α-D-galacturonic acid units joined by glycosidic bonds (α-1,4) and esterified methyl carboxyl groups [23]. Finally, synthesis 3 used *Aloe vera* leaves (*Aloe Barbadensis Miller*), a succulent perennial. The main components of Aloe vera are glycoproteins, anthraquinones, saccharides, and low-molecular-weight substances [24]. The different natural additives used in the MgO syntheses resulted in distinct morphological and surface characteristics. These affected the adsorption of Cu^2+^ ions in wastewater.

## 2. Materials and Methods

### 2.1. Preparation of Magnesium Oxide

The MgO particles were prepared by three different synthesis routes using three sources of polysaccharides as fuel: cassava starch (synthesis 1), citrus pectin (synthesis 2), and *Aloe vera* (synthesis 3). In syntheses 1 and 3, starch extracted from cassava roots and *Aloe vera* leaves in natura were used, respectively. Both organic precursors were harvested from the city of Palmital-Paraná, Brazil. Synthesis 2 used commercial (Dynamic) citrus pectin. The magnesium salt used in the syntheses was magnesium nitrate hexahydrate (Mg(NO_3_)_2_.6H_2_O, 98%, Dynamic). The analytical reagents were of high purity, and all solutions were prepared with deionized water.

The samples were labeled according to the polysaccharide used: starch (MgO-St), citrus pectin (MgO-CP), and *Aloe vera* (MgO-Av).

### 2.2. Synthesis 1—Cassava Starch (MgO-St)

The synthesis method was adapted from Primo et al. [25]. The first step of synthesis 1 was the extraction of starch from cassava roots. Initially, 500 g of natural cassava starch was extracted in 2500 mL of deionized water under mechanical stirring for 3 h. Then, the colloidal suspension was separated from the fibers and used as a base solution. Next, MgO-St oxide was synthesized from starch (300 g) and magnesium nitrate (64 g) and stirred for 20 min.

### 2.3. Synthesis 2—Citric Pectin (MgO-CP)

For synthesis 2, the first step was heating 1500 mL of deionized water to 80 °C and adding 15 g of commercial citrus pectin. This step was adapted from Dalpasquale et al. [23]. Next, this suspension was stirred in a mechanical shaker (800 rpm) for about 1 h to solubilize the citrus pectin. After homogenization of the colloidal suspension, 32 g of magnesium nitrate was added, keeping the agitation and temperature controlled for 3 h, thus forming the MgO-CP material.

### 2.4. Synthesis 3—Aloe vera (MgO-Av)

Synthesis 3 was adapted from Primo et al. (2020) [19]. To obtain the magnesium oxide, gel extracted from *Aloe vera* leaves was used. The gel from the leaves was first removed and processed in a blender (power: 1200 W). The gel was sieved and refrigerated (2 degrees Celsius). Thus, *Aloe vera* gel broth extract with a concentration of 90% was prepared in 100 mL of deionized water. Then, magnesium nitrate (24 g) was dissolved in the aloe extract solution while constantly being magnetically stirred (60 min).

The samples prepared in the different synthesis routes were calcined using the same calcination parameters. The suspensions were calcined in a muffle furnace at 750 °C with a heating ramp of 10 °C·min^−1^ for 60 min. The products obtained were pulverized, sieved using a 250 mm (60 mesh) sieve, and stored in a suitable container for characterization and application.

### 2.5. Adsorption Tests

Adsorption tests were performed to investigate the efficiency of the samples as adsorbents for removing copper from water. The initial and final concentrations of copper metal ions were evaluated for the adsorption test. The adsorption experiments were carried out in flasks of 100 mL containing 50 mL of an aqueous solution of copper sulfate pentahydrate (CuSO_4_.5H_2_O, 99%, Biotec Reagentes Analíticos, Curitiba, Brazil) with initial concentrations of 0.95, 1.6, 3.0, 5.4, 6.4 and 11 g. L^−1^. To this end, 100 mg of each MgO (St, CP, and Av) sample was added to the solutions and kept under continuous shaking in a heating bath at 298 K for a contact time of 60 min. After the adsorption time, the solutions with adsorbent were centrifuged at 1200 rpm for 15 min and the aliquot was separated. Then, the copper ion concentration was monitored by UV–vis spectrophotometer (Shimadzu UV-1800, Kyoto, Japan) at 810 nm.

The amount of Cu^2+^ ions adsorbed at the end of the adsorption experiment and the ion percentage removal (%) by the MgO were calculated by applying Equations (1) and (2), respectively:

(1) $$q=\frac{\left(C_{o}-C_{f}\right)}{m}\cdot V$$

(2) $$\%\mathrm{Removal}=100\;\cdot \frac{\left(C_{o}-C_{f}\right)}{C_{o}}$$

where q is the amount of ions adsorbed by the adsorbent in mg·g^−1^, C_o_ is the initial ion concentration in contact with the adsorbent (mg·L^−1^), C_f_ is the ion concentration (mg·L^−1^) after the batch adsorption process, m (g) is the mass of adsorbent, and *V* is the volume of ion solution (L).

### 2.6. Characterization Techniques

The crystalline structure and phase purity were characterized by X-ray diffractometry (XRD-D2 Phaser; Bruker, Billerica, MA, USA) with a copper cathode (λ = 1.5418 Å), 30 kV potential, 10 mA current, range between 10° and 80° (2θ), and 0.2 °/s increment. Phase Identification from Powder Diffraction, Version 3.4.2, was used with access to the COD database (Crystallography Open Database). The morphology of the MgO particulate samples was examined with a scanning electron microscope (SEM-VEGA 3; TESCAN Brun, Brno, Czech Republic); for the SEM analysis, each sample was dispersed in water, and a drop of dispersion was deposited on an Al sample holder. Before SEM analysis, the specimen surface was coated with a layer of gold. Nitrogen adsorption/desorption isotherms were obtained from the Quantachrome Instruments (Boynton Beach, FL, USA) gas Sorption Analyzer equipment, NOVA 2000 model, using the Quantachrome Novawin software, 11.03 version, the data were measured and analyzed in 24 June 2019. The samples were submitted to vacuum degassing at 200–250 °C for 2 h and 30 min, and the analyses were performed at liquid nitrogen temperature (−196.15 °C). The specific surface areas of the samples were calculated using the multi-point Brunauer–Emmet–Teller (BET) method. Pore volume and mean pore radius were calculated by desorption curve analysis using the Barrett–Joyner–Halenda (BJH) model. The zeta potentials (ζ) of the nanoparticles were determined from their electrophoretic mobilities according to Smoluchowski’s equation [26]. The pH of these nanoparticles was adjusted between 2 and 12 using HCl or NaOH solutions. The measurements were performed on ZETASIZER Malvern equipment, NANO ZS90 model (Worcestershire, UK). For qualitative analysis of MgO synthesized before and after the adsorption of Cu^2+^ ions, energy dispersive spectroscopy (EDS) was performed. The measurements were conducted with an acquisition time of 20 s and an accelerating voltage of 15.0 kV, obtained from Oxford Instruments (Abingdon, United Kingdom), EDS XSTREAM2 model.

## 3. Results

### 3.1. Characterization of Synthesized MgO Particles

#### 3.1.1. X-ray Diffractometry (XRD)

**Figure 1:**
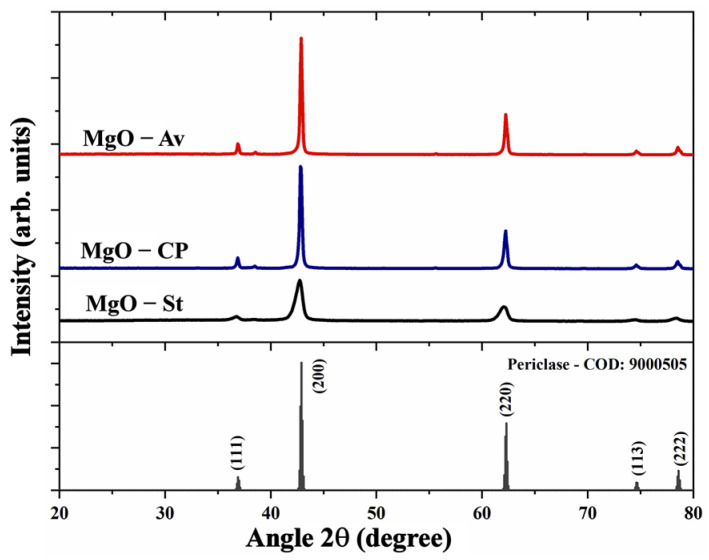
X-ray diffraction of the pattern Periclase [COD: 9000505] and the synthesized samples.

The XRD of the MgO-St, MgO-CP, and MgO-Av samples (Figure 1) showed diffraction peaks at 36.86°, 42.88°, 62.30°, 74.71°, and 78.73°, respectively. These diffraction peaks corresponded to the (111), (200), (220), (113), and (222) planes, respectively, which was in good agreement with the standard pattern of face-centered cubic (FCC) and periclase phase of MgO (COD: 9000505) with a space group of Fm-3 m [18]. Furthermore, the diffractograms (Figure 1) were similar, suggesting that the fuel used did not affect the crystal structure of the MgO particles. Nevertheless, the diffractogram peaks of the oxide synthesized with starch fuel (MgO-St) showed larger (57% wider, approx.) widths at their average height compared to the peaks of the MgO-CP and MgO-Av diffractograms, suggesting a smaller crystallite size [27,28].

#### 3.1.2. Morphological Analysis (SEM)

**Figure 2:**
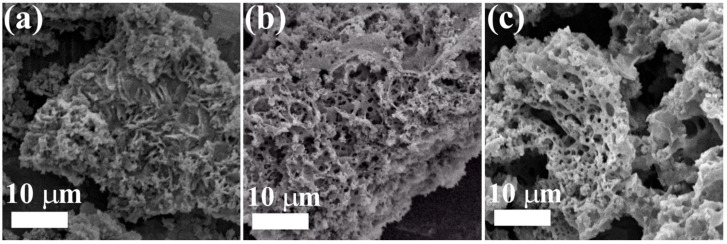
SEM images of the synthesized MgO samples MgO-Av (**a**), MgO-CP (**b**), and MgO-St (**c**).

The scanning electron microscopy micrographs of the synthesized powders using various fuels are shown in Figure 2a–c. Figure 2a illustrates the morphology of the MgO-Av sample. It was composed of interconnected nanosheets [29]. Figure 2b shows the porous morphology of the MgO-CP sample. Finally, the morphology of the MgO-St sample (Figure 2c) showed sponge-like characteristics, with non-regular pores and holes along the entire surface. These pores were reported to be associated with releasing gases from the organic fuel when calcined [30].

#### 3.1.3. Textural Properties (BET and BJH Isotherms)

**Figure 3:**
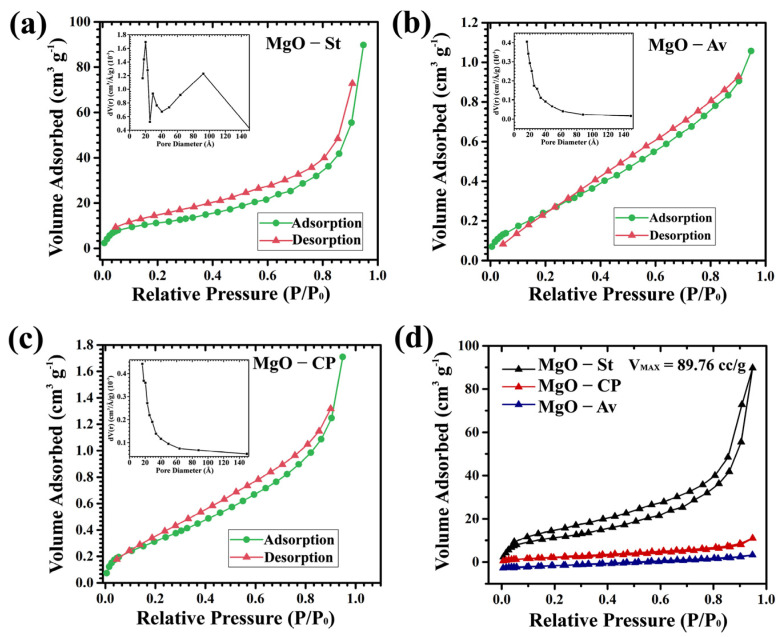
Adsorption and desorption isotherms with pore size distribution (inset) of MgO samples: (**a**) starch, (**b**) *Aloe vera*, (**c**) citrus pectin, and (**d**) comparison of the maximum volume of adsorbed N_2_.

The samples’ N_2_ adsorption–desorption isotherms (Figure 3) were calculated using the multi-point Brunauer–Emmet–Teller (BET) method. In addition, the pore volume and radius were calculated by analyzing the desorption curve using the Barrett–Joyner–Halenda (BJH) model. The isotherms were type IV according to the IUPAC classification, indicating completely reversible adsorption–desorption isotherms associated with mesopores for MgO-St sample (pore diameter between 20–50 Å) and micropores for MgO-Cp and MgO-Av samples (<20 Å) (Table 1) [31]. The MgO-St sample (Figure 3a) showed an H4-type hysteresis loop often related to materials with slit-like pores between particles, indicating that pore filling occurred [32].

The MgO-St surface area, pore volume, and diameter (Table 1) were larger than those of the other two materials, showing wider micropores and possibly narrow mesopores (between 20–500 Å) [31]. The MgO-Av (Figure 3b) and MgO-CP (Figure 3c) samples also showed an H4-type hysteresis loop, with a smaller surface area and pore volume, which implied large pores, however, this was not as profound as for the MgO-St sample. The maximum volume was due to N_2_ being adsorbed for forming complete gas monolayers on the sample surface at partial pressure [32]. The MgO-St sample showed an increase of ~22.7% in the pore radius, suggesting a larger pore volume (0.131 cm^3^/g) and a larger surface area (41 m^2^/g). These textural aspects indicated a greater adsorption capacity of N_2_ gas. The maximum volume adsorbed on the surface of each oxide presented values in cc/g (Figure 3d). The MgO-St sample showed the largest adsorbed volume (V_MAX_ = 89.76 cc/g), while the adsorbed volume for the MgO-Av (V_MAX_ = 6.44 cc/g) and MgO-CP (V_MAX_ = 11.01 cc/g) samples were much smaller (Table 1).

#### 3.1.4. Zeta Potential (ζ) vs. pH

**Figure 4:**
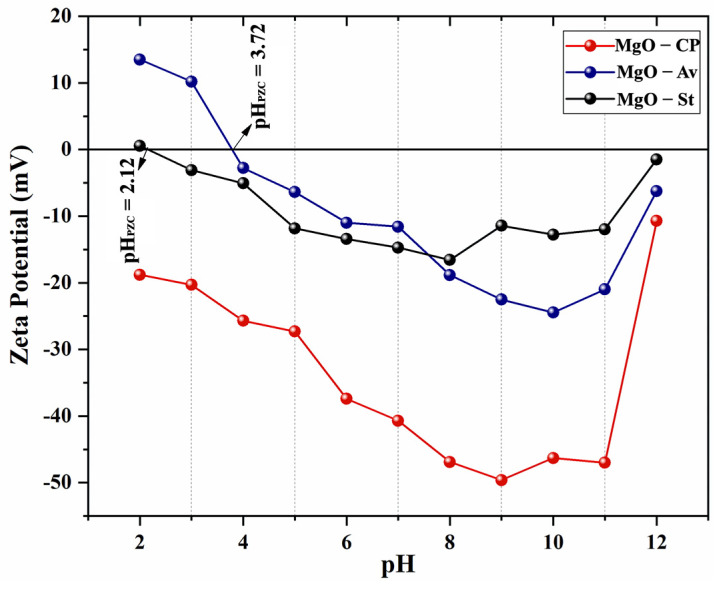
Zeta potential as a function of pH and point of zero charge (pH_PZC_) for the MgO-St, MgO-CP, and MgO-Av samples.

Figure 4 shows the changes in the zeta potential (ζ) of the samples during the NaOH titration for the synthesized oxides. The zeta potential allows the evaluation of the chemical stability of particles in a colloidal state [19]. Figure 4 shows that with the addition of acid solution, i.e., the addition of H^+^ ions, the initial dissociation of MgO occurred, which results in the approximation of H^+^ ions and the dissociation in Mg^2+^ and OH^−^. This step decreased the zeta potential of the MgO due to the neutralization of the surface charge. At low pH, the number of positive ions increased, preferably at the magnesium oxide surface, increasing the zeta potential. The pH of the solution decreased with the increase in this charge, reducing the tendency of agglomeration [33].

The ζ potential of the MgO-CP sample during the pH titration range of 6–11 (Figure 4) was less than −30 mV, indicating that in this range, its particles were highly stable in the suspension, which prevented the particles from flocculating [34]. Therefore, the profile of the zeta potential for the MgO-CP sample standing out as the zero-charge point (pH_PZC_) was not observed. This probably occurred due to the adsorption of negative ionizable groups on the MgO particles, which decreased the mobility, reduced the surface electric charge of MgO (positive), and consequently decreased the zeta potential and medium fluidity [33].

The samples synthesized with starch and *Aloe vera* showed positive values when the pH < 3; for the MgO-Av sample, the surface charge showed a positive ζ potential value equal to + 1.24 mV, and for the MgO-St sample, it was + 14 mV. With increasing the pH, the zero-charge point (pH_PZC_) was reached at 2.12 for MgO-Av and 3.72 MgO-St.

The pH values of each solution were measured as soon as it was prepared, without any external acidic or basic modifications. The pH values of each solution are shown in Table 2.

The impact of the pH on Cu^2+^ removal was appreciable, while the interaction between negative and positive ions played a determining role in the adsorption on the adsorbent surface. This was due to changes not only to the active sites on the MgO surface, which could coordinate the copper metal but also to the solubility of Cu^2+^ in the aqueous solution [12]. Magnesium oxide is a highly alkaline particle, and therefore sorption of Cu^2+^ onto MgO surfaces can be achieved by chemical sorption and/or ion exchange [35]. In the studies by Ismail et al. (2022) [12], they also used MgO as an adsorbent to remove Cu^2+^ in a solution with a concentration of 90 g·L^−1^ in a pH range from 1 to 9, obtaining the best removal results in solutions at pH 3 and 5. This is because the minimal adsorption at pH 2.0 may be because there are more protons at lower pH available to protonate active groups on the surface and compete with the metal ions in the solution. At higher pH values, the lower number of H+ and higher number of negatively charged ligands results in greater copper adsorption [36]. As the pH increases, the adsorbent surface becomes more and more negatively charged, and therefore the adsorption of positively charged Cu^2+^ species is quite favorable. According to previous research [36,37], Cu^2+^ is only available in the divalent ion form at pH values below 5.0. At pH 6.0 and higher, various neutral hydrolysis species can be found.

## 4. Copper Ion Removal by MgO as Adsorbent

### 4.1. Adsorption Isotherms

**Figure 5:**
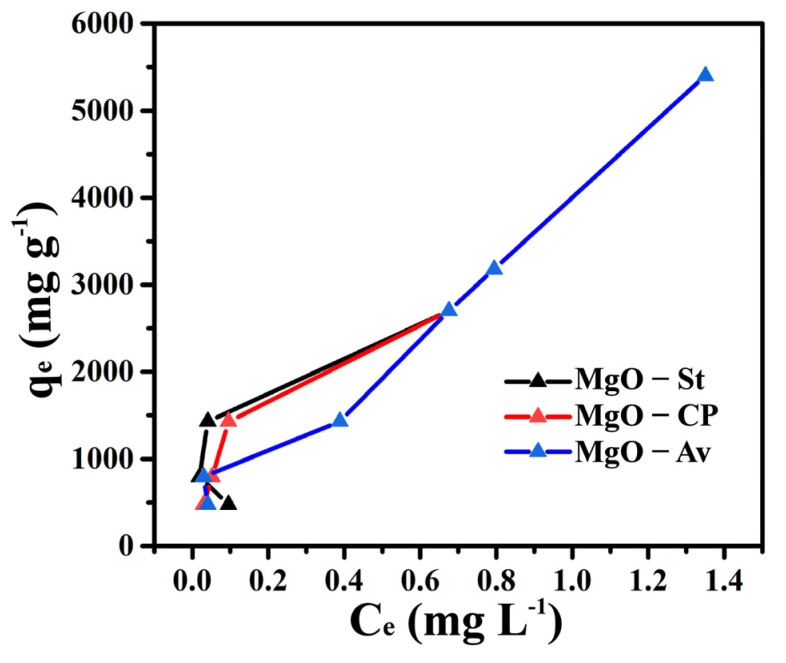
Experimental point curves for the adsorption of Cu^2+^ ions onto MgO-St, MgO-Av, and MgO-CP at 298 K.

The removal of Cu ^2+^ ions onto the surfaces of the MgO-St, MgO-Av, and MgO-CP oxides was effectively reached, even at high initial concentrations. Figure 5 shows that the adsorption capacity of the synthesized MgO is related to the initial concentration of copper. The results reveal that the more is copper present in the solution, the stronger is the interaction between the copper ions with the adsorbent, and the more susceptible to exchange on the MgO surface [11]. Similar results were observed in [38], using mesoporous–macroporous MgO (HMMM) via a calcination process, to remove uranium from an aqueous solution in high concentration. The maximum adsorbed amount of all the magnesium oxides was obtained at a very close value of the equilibrium concentration of Cu^2+^ ion in solution, also observed in Figure 5; this can be explained by the fact that the structure of all the materials was the same as observed in the DRX results (Figure 1). The sites for the adsorption of Cu^2+^ ions were available in a very similar way; this made the amount of Cu^2+^ ions adsorbed practically equal in higher concentrations, as observed in Figure 5.

The data for the adsorption of Cu^2+^ ions onto the MgO-St, MgO-Av, and MgO-CP oxides were adjusted according to the Langmuir, Freundlich, and Temkin isotherm models and their correlation parameters are presented in Table 2. For the practical design of adsorption systems, the correlation between theoretical data and empirical equations is significant [39]. The Langmuir model is applicable for systems with the assumption of ideal homogeneous surface adsorption. Therefore, the Freundlich model fits better for a heterogeneous system. The Langmuir isotherm in linear form is usually given as (Equation (3)):

(3) $$\frac{C_{e}}{q_{e}}=\frac{1}{{\mathrm{qL}}_{\max}\frac{C_{e}}{q_{\max}}}$$

where q_e_ (mg·g^−1^) is the amount of ions adsorbed per unit mass of MgO at equilibrium, K_L_ (L·mg^−1^) is the Langmuir constant related to the affinity of the binding sites, and q_max_ (mg·g^−1^) is a parameter associated with the maximum amount of Cu^2+^ per unit weight of MgO.

The linearized form of the Freundlich isotherm is given as (Equation (4)):

(4) $$\ln q_{e}=\ln K_{F}+\frac{1}{n}\ln C_{e}$$

where K_F_ (mg L^−1^) is the Freundlich constant, n is a parameter related to the adsorption intensity and system heterogeneity, and K_F_ and n are the Freundlich constants found from the intercept and slope of the straight line of the plot ln_qe_ versus ln_Ce_.

The Temkin model isotherm ignores extremely low and very high concentrations and assumes a linear rather than a logarithmic decrease in the heat of adsorption [40]. This model assumes a uniform distribution of the bonding energy up to some maximum bonding energy [40,41]. It can be expressed as (Equation (5)).

(5) $$q_{e\;}=\frac{\mathrm{RT}}{b}\ln C_{e\;}+\frac{\mathrm{RT}}{b}\ln K_{T}$$

where b is the Temkin constant (J·mol^−1^), T is the absolute temperature (K), R is the gas constant (8.314 J·mol^−1^·K^−1^), and A is another Temkin isotherm constant (L·g^−1^).

**Figure 6:**
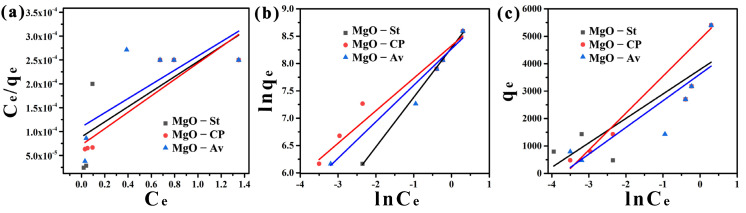
The linear form of the Langmuir (**a**), Freundlich (**b**), and Temkin (**c**) adsorption isotherms for the adsorption of Cu ^2+^ ions onto MgO-St, MgO-Av, and MgO-CP.

The correlation coefficient (R^2^) obtained by the Freundlich (R_F_^2^) isothermal model was better fitted than the Langmuir (R_L_^2^) and Temkin (R_T_^2^) models for the MgO-St and MgO-CP samples, as observed in Figure 6 and Table 3. However, the adsorption of ions onto the MgO-Av sample was well fitted for the Langmuir and Temkin models (Table 3). When observing the correlation coefficient (R^2^), better-fit results were obtained for the Freundlich isotherm model (R_F_^2^ = 0.989 > R_T_^2^ = 0.987). Therefore, as the Freundlich was the best-fitted model, the adsorption process of Cu^2+^ ions onto the MgO-St, MgO-Av, and MgO-CP samples consisted of multilayer adsorption, as this isotherm model is an empirical model and is not limited to a monolayer process [40,41].

In the study of Huang et al. (2021) [41], a 2D carbon ribbon (MIL-53/C) and a nitrogen-doped carbon ribbon (MIL-53-N/C) were successfully synthesized using 2D-MOFs, and they were used in the removal of high fluoride concentrations in water, and the adsorption isotherm results also showed a better fit to the Freundlich model for the adsorption of fluoride by MIL-53/C. The adsorption was a multilayer process, and the maximum adsorption capacity was recorded at 299.17 mg·g^−1^ for the same sample.

The Freundlich isotherm was also best described in the study of Inyinbor et al. (2016) [40], in which the researchers investigated the uptake of Rhodamine B dye onto *Raphia hookeri* fruit epicarp, obtaining an R^2^ value of 0.9969, suggesting an adsorption process that was not onto a uniform site but rather multilayer adsorption.

One of the other parameters that can be considered in the Freundlich model is the value of n. This factor determines the affinity of the process, that is, the process is considered to involve chemisorption (n < 1) or physisorption (n > 1). From Figure 6 and considering the parameters in Table 2, the Freundlich plot resulted in 1/n values of 1.103, 1.683, and 1.500 for the MgO-St, MgO-Av, and MgO-CP samples, respectively. These values indicated that physisorption occurred [42]. When a physisorption adsorption process is considered, in general, a van der Waals bond exists between the adsorbate and adsorbent, and the physisorption process is postulated to include the existence of multilayer adsorption onto the adsorbent, which is line with the Freundlich model.

The maximum adsorption capacity (q_max_) obtained by the Langmuir isotherm model was calculated, and values of 6331.117, 5831.244, 6726.623 mg··g^−1^ were obtained for the MgO--St, MgO--Av, and MgO--CP adsorbents, respectively, at a high concentration of Cu^2+^ ions. These values were comparable to the studies reported in the literature, and are significantly higher than those reported, as shown in Table 4. In addition, the maximum capacity of Cu^2+^ adsorption was highlighted when compared with the adsorption capacity of the other magnesium oxides due to the ion exchange process that occurs when MgO is added into different concentrations of Cu^2+^ ion solutions.

Moscofian and Airoldi (2008) [43] synthesized a magnesium phyllosilicate modified with 2-aminophenyldisufide for the adsorption of Cu^2+^, Pb^2+^, and Cd^2+^ ions. The results showed that the adsorption process of all the ions investigated was better fitted by the Langmuir isotherm model, whose maximum adsorption capacity reached 208.44 mg·g^−1^ for the adsorption of Cu^2+^ ions. In the study of Ciesielczyk et al. (2017) [44], Lignin/MgO-SiO_2_, a hybrid sorbent, was applied to the adsorption of galvanic waste solutions of copper (II) ions, and the adsorption process was well fitted by the Langmuir model, obtaining an R^2^ of 0.982 and adsorption capacity equal to 83.98 mg·g^−1^. Xu et al. (2019) [39] investigated electrospun SiO_2_–MgO hybrid fibers as adsorbents of Pb^2+^ and Cu^2+^ ions. The results showed that the adsorption of Cu^2+^ ions onto the electrospun SiO_2_–MgO hybrid fibers at 298 K obtained an adsorption capacity equal to 492.96 mg·g^−1^. In addition, the Langmuir isotherm was well fitted in this adsorption process. Recently, Ismail et al. (2022) [12] investigated the impact of Cu^2+^ ion removal on MgO nanostructures. To manufacture the MgO nanoparticles, a thermal pyrolysis process was used. The regression coefficient for the Langmuir isotherm model for the adsorption of Cu^2+^ ions onto the MgO nanoparticles was 0.9960, and the highest adsorption capability of Cu^2+^ was 546.45 mg·g^−1^.

### 4.2. Adsorption Mechanisms

#### 4.2.1. Energy Dispersive Spectroscopy (EDS)

Heavy metal adsorption has four mechanisms: precipitation, ion exchange, complexation of functional groups, and interaction between metal ions and π electrons. The samples were analyzed by energy dispersive spectroscopy (EDS) after Cu^2+^ adsorption to understand the occurring mechanism of copper ion adsorption (Table 5). The analyses were performed before adsorption (MgO-St, MgO-CP, and MgO-Av) and after adsorption at the lowest (0.95 g·L^−1^) and highest concentration (11.0 g·L^−1^).

The EDS data were analyzed before and after the adsorption of copper ions (Table 4). Before the Cu^2+^ ion removal process, a higher percentage of Mg ions was observed. The Mg percentage decreased as the copper concentration increased. Therefore, higher concentrations of Mg^2+^ were released during the Cu^2+^ adsorption. From the EDS data, we can suggest the occurrence of the cation exchange mechanism. In which the Mg ions that are part of the MgO crystalline network are replaced by Cu^2+^ cations in the network. This releases into the water magnesium ions that are atoxic and not harmful to the environment [12]. In general, as a solid-liquid interfacial process, the adsorption of ions onto adsorbents is initiated by electrostatic attraction and can also be followed by ion exchange, which usually occurs between the hydroxyl groups on the surface and the anions [45] (such as copper ions). The possible reaction promoted by MgO can be explained by Equations (6)–(8) [45].

(6) $${\mathrm{MgO}}_{\left(s\right)}+{\;H}_{2}\;O\;\to \mathrm{Mg}{\left(\mathrm{OH}\right)}_{2}$$

(7) $${\mathrm{MgO}}_{\left(s\right)}+{\;\mathrm{CuSO}}_{4}\;\to \mathrm{CuO}+{\mathrm{Mg}}^{2+}+{\mathrm{SO}}_{4}^{2-}$$

(8) $$\mathrm{Mg}{\left(\mathrm{OH}\right)}_{2}+{\;\mathrm{CuSO}}_{4}\;\to \mathrm{Cu}{\left(\mathrm{OH}\right)}_{2}+{\mathrm{Mg}}^{2+}+{\mathrm{SO}}_{4}^{2-}$$

Table 5 presents the percentage by mass of the elements in the samples. Among the precursors, the *Aloe Vera* sample contained the highest percentage of magnesium. Considering the lowest concentration tested, the best material in the adsorption of copper was MgO-Av, while at the highest Cu^2+^ concentration tested, the highest percentage was adsorbed by the MgO-St sample. This may have occurred due to the surface area, pore volume, and diameter being higher than the other samples, as seen in the BET. The presence of sulfur originated from the precursor salt used for the tests.

#### 4.2.2. Fourier Transform Infrared Spectroscopy (FTIR)

**Figure 7:**
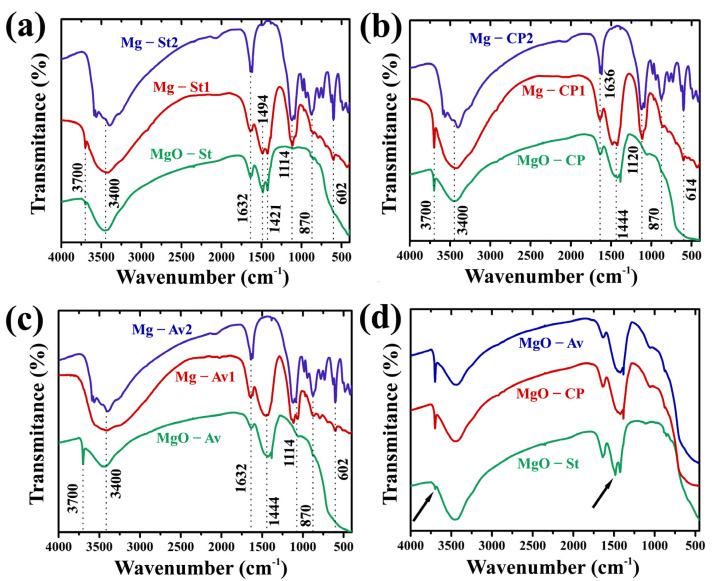
FTIR spectra of MgO before and after Cu^2+^ adsorption for MgO-St (**a**), MgO-CP (**b**), and MgO-Av (**c**) with an increased Cu^2+^ concentration, and comparison between the three synthesized oxides (**d**).

To further explore the changes in the functional groups on the surface of the adsorbents before and after the copper adsorption in the two different concentrations of adsorption (0.95 and 11 g·L^−1^), we performed FTIR spectroscopy as shown in Figure 7. Figure 7a–c shows a narrow and intense band at 3700 and 3400 cm^−1^, which was attributed to the vibrational stretching of the free -OH ions of the Mg(OH)_2_, which were generated by the hydration of MgO [46]. These hydroxyl groups would be active adsorptive sites, and the hydrated Cu^2+^ ions can react with the hydroxyl groups electrostatically [47]. In addition, the adsorbed hydrated Cu^2+^ ions could partially hydrolyze and form Cu–OH, leading to the formation of Cu–O–Cu on the pore walls. Therefore, multilayer adsorption could take place on the porous MgO, exhibiting strong adsorptive performance and Freundlich-type adsorptive behavior [47], corroborating the results obtained for the synthesized oxides. The band at 3700 cm^−1^ was exhibited after adsorption for the lower concentration of copper by the MgO-St (Mg-St1, Figure 7a) and MgO-CP (Mg-CP1, Figure 7b) oxides. This OH stretching vibration band at 3700 cm^−1^ was changed in intensity after adsorption at the lower concentration, showing that the copper ions, Cu^2+^, interacted with the OH in Mg(OH)_2_ to form a Mg–O–Cu bond [12]. This result was confirmed by the shifting of the MgO vibration band located at 870 cm^−1^ for the lower and higher concentration of Cu^2+^, and the intensity of it was increased with the higher concentration of Cu^2+^ [12,48]. However, for Mg-Av1 and Mg-Av2 (Figure 7c), Mg-St2 (Figure 7a), and Mg-CP2 (Figure 7b), no surface hydroxyl groups were detected around 3700 cm^−1^, suggesting that the surface hydroxyl groups were too active to react with CO_2_ in air and to subsequently form surface carbonate-like species [48]. The band at around 1632–1636 cm^−1^ for all the samples corresponded to the O–H bending mode, which is characterized by the bending vibration of the -OH group of the physiosorbed water molecules [46,49]. The bands at 1444 and 1114 cm^−1^ were attributed to the asymmetric and symmetric stretching of a unidentate carbonate on the surface, respectively [12,48]. The existence of carbonate ions could be estimated as the absorption of carbon dioxide gas from the atmosphere [50]. These bands explained the percentage of carbon identified by EDS in each oxide before and after adsorption. In addition, the intensities of the FTIR bands of the surface carbonate-like species were also changed after the Cu^2+^ sorption. That is to say that the surface unidentate carbonate species may have contributed to the Cu^2+^ removal mechanism [48]. Rhatingan and Nolan (2018) [51] presented a DFT study of TiO2 anatase (101) modified with SnO and MgO nanoclusters, with specific Sn_4_O_4_ and Mg_4_O_4_ compositions. The authors explained that point defects, such as oxygen vacancies, aid the chemistry on the metal oxide surface by providing sites for the adsorption and activation of molecules such as H_2_O and CO_2_. The results revealed that Cu^2+^ had a strong absorption band after being removed at 602–614 cm^−1^, which was identified as the metal–oxygen (Cu–O) bond bending mode [50], suggesting that ionic exchange has occurred between Mg^2+^ and Cu^2+^, forming this bond, associating with Equation (6) and corroborating with the EDS data. In comparing the three oxides synthesized before the copper removal (Figure 7d), a few differences were identified in the adsorption bands. The MgO-St oxide showed less intensity in the band at 3700 cm^−1^ and a small band at 1494 cm^−1^, corresponding to the O–H bending mode [46,49].

## 5. Conclusions

In summary, the MgO samples synthesized using polysaccharides as the reaction fuel showed a superior porosity and surface area compatible with porous materials that can remove metal ions, such as Cu^2+^, from wastewater. The pores evidenced by SEM were from the gases released by the fuels used in the syntheses. The synthesized oxides showed the same crystalline structure, although they were synthesized from different polysaccharides. According to the Langmuir, Freundlich, and Temkin adsorption models, the adsorption patterns were fitted to the isotherms. For the Langmuir model, the MgO-CP material showed a maximum adsorption capacity of 6726.623 mg·g^−1^, and the other samples also showed excellent results, with values of q_max_ of 6331.117 and 5831.244 mg·g^−1^ for MgO-St and MgO-Av, respectively. Before and after the adsorption tests, the oxides and the solid samples after the adsorption at concentrations of 0.95 and 11 g·L^−1^ were subjected to EDS analysis, which showed that as the insertion of Cu ions into the MgO structure occurred, Mg^2+^ ions were reduced. The same samples were also analyzed by FTIR, which showed bands referring to Mg–O–Cu bonds (870 cm^−1^) and a Cu–O bond band at 602–614 cm^−1^, confirming the adsorption of copper by the MgO-St, MgO-CP and MgO-Av oxides. Due to their high adsorption efficiency, the synthesized oxides with different polysaccharides are potential adsorbents for heavy metal removal.

## Figures and Tables

**Table 1:** Textural properties of the synthesized MgO.

Samples	Surface Area (m^2^/g)	Pore Volume (cm^3^/g)	Pore Diameter (Å)	Volume (cc/g)
MgO-St	43.93	0.130	20.06	89.76
MgO-CP	7.01	0.015	16.29	11.01
MgO-Av	6.63	0.009	16.29	6.44

**Table 2:** pH values of the solutions before adsorption.

Adsorption Solutions (g·L^−1^)	pH
0.95	3.77
1.6	3.54
3.0	3.27
5.4	3.11
6.4	3.07
11	2.88

**Table 3:** Parameters of the Langmuir, Freundlich, and Temkin isotherms for adsorption of Cu ^2+^ ions onto MgO-St, MgO-Av, and MgO-CP.

Samples	Langmuir			Freundlich			Temkin		
	*K_L_* (L·mg^−1^)	q_max_ (mg·g^−1^)	R_L_^2^	*K_F_* (L·g^−1^)	1/n	R_F_^2^	*K_T_* (L·g^−1^)	b (J·mol^−1^)	R_T_^2^
MgO-St	1.779	6331.117	0.589	3974.443	1.103	0.999	76.831	2.777	0.725
MgO-Av	2.386	5831.244	0.817	4125.036	1.683	0.989	40.953	1.840	0.987
MgO-CP	1.356	6726.623	0.547	3895.705	1.500	0.991	44.904	2.546	0.733

**Table 4:** Comparison of copper’s adsorption capacities (mg·g^−1^) using magnesium oxide as adsorbent.

Adsorbent	Adsorption Capacities q_max_ (mg·g^−1^)	References
Magnesium phyllosilicate modified with 2-aminophenyldisufide	208.44	(Moscofian and Airoldi, 2008) [43]
Lignin/MgO-SiO_2_	83.98	(Ciesielczyk et al., 2017b) [44]
Electrospun SiO_2_-MgO hybrid fibers	493.00	(Xu et al., 2019) [39]
MgO nanostructures	546.45	(Ismail et al., 2022) [12]

**Table 5:** Compositional chemical analysis data by EDS (% element) for the samples before and after adsorption at lowest (Mg-St1, Mg-CP1, and Mg-Av1) and highest concentrations (Mg-St2, Mg-CP2, and Mg-Av2).

Samples	Mg%	O%	C%	Cu%	S%
MgO-St	37.01	51.00	11.99	-	-
Mg-St1	11.66	47.97	19.75	18.24	2.38
Mg-St2	0.44	41.66	8.34	42.91	6.66
MgO-Av	50.06	44.86	5.08	-	-
Mg-Av1	16.43	44.78	11.66	23.99	3.14
Mg-Av2	1.04	41.11	11.14	39.62	7.09
MgO-CP	41.55	48.41	10.04	-	-
Mg-CP1	12.75	48.50	19.11	17.60	2.04
Mg-Cp2	0.20	40.65	13.47	39.44	6.25

## Data Availability

The data presented in this study are available on request from the corresponding author.
